# Feeding Habits of Introduced Black Rats, *Rattus rattus*, in Nesting Colonies of Galapagos Petrel on San Cristóbal Island, Galapagos

**DOI:** 10.1371/journal.pone.0127901

**Published:** 2015-05-18

**Authors:** Marjorie Riofrío-Lazo, Diego Páez-Rosas

**Affiliations:** 1 Galapagos Science Center, Universidad San Francisco de Quito (USFQ), San Cristóbal Island, Galápagos, Ecuador; 2 Dirección Parque Nacional Galápagos, Unidad Técnica Operativa San Cristóbal, San Cristóbal Island, Galápagos, Ecuador; Institute of Ecology, GERMANY

## Abstract

Introduced rodents are responsible for ecosystem changes in islands around the world. In the Galapagos archipelago, their effects on the native flora and fauna are adverse, including the extinction of endemic rodents in some islands and the reduction in the reproductive success of the Galapagos petrel (*Pterodroma phaeopygia*) in its nesting zones. Understanding the feeding behavior of introduced rodents and their trophic interactions with native and non-native species on islands, can assist in the design of management strategies and conservation plans of invasive and endemic species respectively. Four petrel nesting colonies were monitored during June 2013 on San Cristóbal Island (El Plátano, El Junco, San Joaquín, and La Comuna). The feeding habits of black rats were evaluated by analyzing stomach contents and stable isotopes in hair. Three species of introduced rodents were captured. *R*. *rattus* was the most abundant at all sites (n=43, capture success (CS) = 55.8%), followed by the house mouse, *Mus musculus* (n = 17, CS = 37.8%), and the Norwegian rat, *R*. *norvegicus* (n = 4, CS = 4.5%), captured only at La Comuna. The omnivorous black rat ate mostly plants (98%) and arthropods (2%). Intact seeds of *Miconia robinsoniana* were the main food at all sites (relative abundance=72.1%, present in 95% of the analyzed stomachs), showing the black rats’ possible role in the archipelago as endemic seed dispersers. There was no evidence of petrel’s intake; however, its possible consumption is not discarded at all. The δ^15^N and δ^13^C analysis corroborated the primarily herbivorous diet of black rats. The isotopic signatures of the three rodent species reflect the inter- and intra-specific differential use of food resources. Black rat showed a wider diet in La Comuna, which was related to a lower availability of its primary prey and its ability to adapt to the available resources in its habitat.

## Introduction

Introduced rodents on islands are associated with altered terrestrial communities and modified ecosystem properties [1,2]. Rodents directly impact terrestrial communities by feeding on plants and animals [3,4,5] and are a significant threat to island bird populations [6]. For instance, the reduction in the reproductive success of the Galapagos petrel *Pterodroma phaeopygia*, an endemic and critically endangered seabird, is related to egg and chick predation by the black rat, *Rattus rattus* [7,8], and it has been considered the primary cause of nest failure.

For conservation purposes the quantification of ecological relationships, especially prey selection by predatory rats, is a critical first step in the design of mitigation plans [9]. The effects of introduced small mammals on insular ecosystems is difficult to assess [10] primarily because of interactions with other co-introduced species [11].

In the Galapagos Islands, three species of invasive rodents are present: the black rat, the Norwegian rat (*R*. *norvegicus*) and the house mouse (*Mus musculus*); which on several islands have become sympatric [12].

Studies on the feeding behavior of black rats in different environments of the Galapagos Islands have demonstrated that this species is a highly selective consumer, despite eating a wide range of plants and animals, the same 2–4 items comprise the diet of most rats [3,13].

House mouse is an introduced omnivorous rodent which is clearly able to adapt to local resource availability. Although it consumes plants, is more carnivorous than the black rat [14,15,16]. In Galapagos it has been demonstrated to eat more plant material than animal material [17].

The Norwegian rat is omnivorous and usually eats animal material [18]. This rodent mainly inhabits urban zones, but it has also been captured in the highlands of Santa Cruz Island, Galápagos [19]. There is no record of Norwegian rats in the petrel nesting habitat in San Cristóbal, however is probable its presence due to human migration to the highlands.

The analysis of stomach contents only represents the food consumed during the sampling event [13]. Nevertheless, stable isotopes measured in various tissues (blood, hair, feathers, teeth, etc.) allow the inference of the feeding habits of species in different time scales according to the tissue in question. Isotope analysis provides information about the level and trophic amplitude in the case of δ^15^N [20,21], and about the feeding habitat or energy source in the case of δ^13^C [22]. Through obtaining information about the assimilated food, analysis of stable isotopes lowers the uncertainty in the estimated diet of species measured with traditional methods.

In the Pacific, the black rat’s diet has been evaluated both with traditional methods and with stable isotopes [23,24,25], and in general, these studies confirm that the black rat is highly omnivorous, consuming a wide range of plants, invertebrates, vertebrates and mushrooms [26].

The present study identified the rodent species that inhabit four main petrel nesting colonies on San Cristóbal Island, as well as their relative abundance, through ecological monitoring of these zones during June 2013. Analyses of stomach contents and stable isotopes in hair were used to identify the diet of the rodents and to determine possible differences between populations. The coexistence of black rats with other introduced rodent species found in the study zone is discussed. These results provide insights into the trophic ecology of black rats and their possible impacts on particular native flora and fauna species in humid highlands of San Cristóbal Island, Galapagos.

## Methods

### Ethics statement

This research was performed as part of a petrel population monitoring and control of introduced rodents’ species by the Galapagos National Park (GNP) under the research permit No PC: 36–13 approved and supported by this institution. This research was carried out following the protocols of ethics and animal handling approved by the GNP and the Galapagos Science Center, Universidad San Francisco de Quito.

### Study area and sample collection

**Fig 1:**
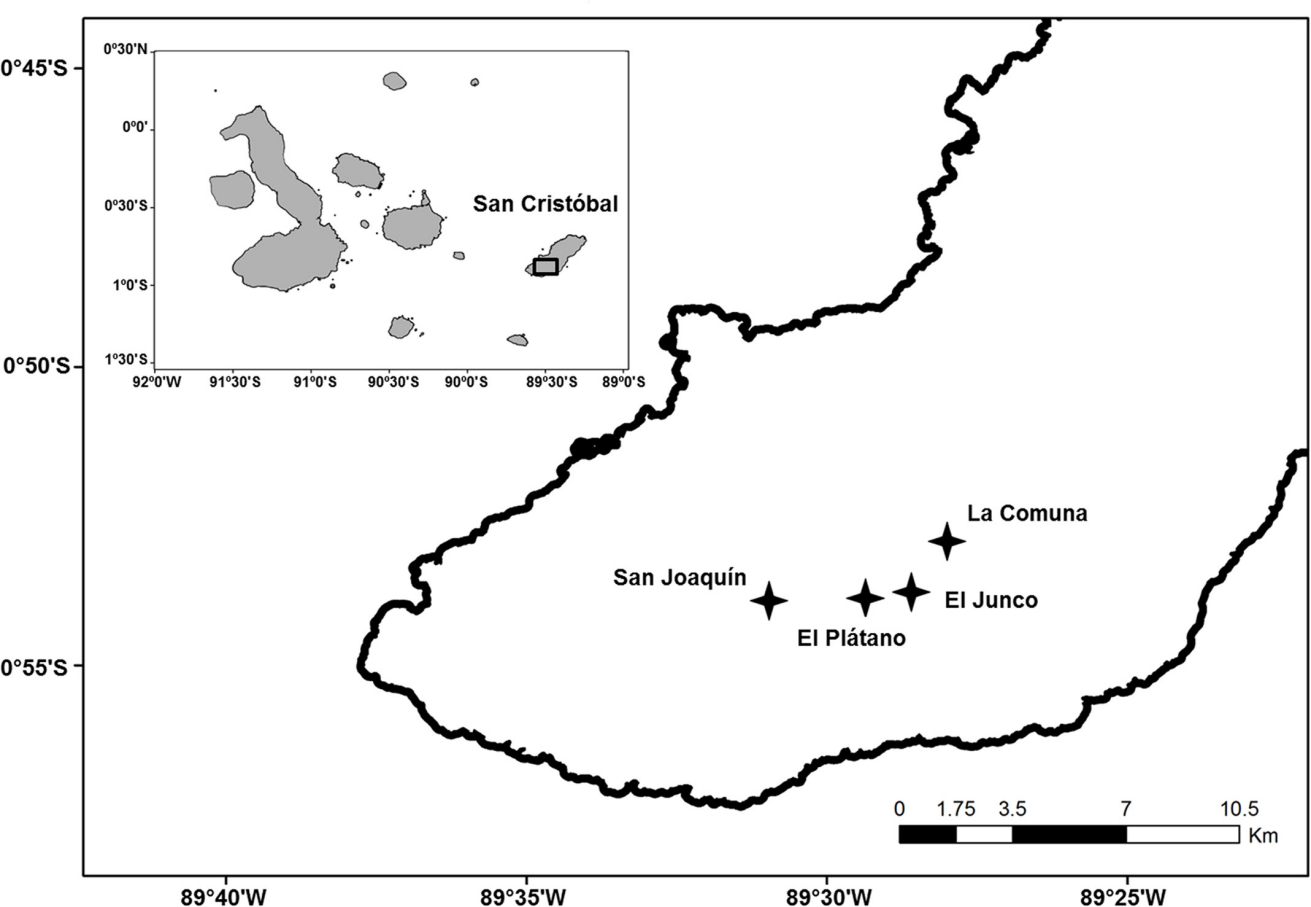
Map of San Cristóbal Island, Galapagos, showing the study sites: San Joaquín (0°53′90″ S, 89°30′93″ W), El Plátano (0°53′88″ S, 89°29′21″ W), El Junco (0°53′80″ S, 89°28′51″ W) and La Comuna (0°52′97″ S, 89°28′00″ W).

The study was conducted in four Galapagos petrel nesting colonies on San Cristóbal Island: El Junco (0°53′80″ S, 89°28′51″ W; elevation: 620 m), El Plátano (0°53′88″ S, 89°29′21″ W; elevation: 569 m), La Comuna (0°52′97″ S, 89°28′00″ W; elevation: 486 m) and San Joaquín (0°53′90″ S, 89°30′93″ W; elevation: 637 m), from 2 to 28 June 2013 (Fig 1). El Junco is inside the protected area of the Galapagos National Park, while the other sites are located inside the private agricultural lands.

The vegetation of the area, primarily from zones with elevation higher than 600 m, includes patches of the endemic shrub Galapagos Miconia (*Miconia robinsoniana*), ferns, terrestrial and epiphytic liverworts and areas covered with grasses locally known as “pampas” [27]. Introduced species such as raspberry (*Rubus niveus*) and guava (*Psidium guajava*) are common in these zones, and domestic animals such as cattle, horses and pigs are frequently in the proximity of nests. Petrel nests are mostly located along ravines, in areas of dense vegetation cover formed by Galapagos Miconia and a wide variety of native ferns [27].

Traps to capture live rodents, 30 Tomahawk-type for rats and 20 Sherman-type for mice, were placed randomly at each site covering the petrel nesting habitat. The trapping area was 3.5 ha in El Junco, 5.9 ha in El Plátano, 4.8 ha in La Comuna and 5.3 ha in San Joaquín. The total nests recorded inside the trapping area was 10 in El Junco, 8 in El Plátano, 24 in La Comuna and 19 in San Joaquín. The distance between traps was 15–20 m and approximately 5 m from nests. Each trapping event consisted of a night without bait (open traps) and three capture nights. A mix of peanut butter and oats was used as bait. The traps were activated at sunset and checked early the following morning. The presence of rodents in traps, although they were not captured, was recorded by detection of scats, urine and traces of bait eaten. Captured rats were anaesthetised using acetone then killed by cervical dislocation. In the laboratory, stomachs were removed and preserved in 70% alcohol.

### Analysis of stomach contents

Stomach contents were sieved under running water with a sieve No. 60 with 0.25 mm mesh size. The found structures were placed on a Petri dish and examined with a stereomicroscope. Stomachs that contained excessively digested material were discarded [28]. The items found in the stomachs were ordered by food category, and these were identified to the species level when possible. Specialized guides were used for identification [29]. The main types of food considered were as follows: (1) Plants: fruits, seeds, leaves, flowers, woody and herbaceous stems, roots and rhizomes; (2) Invertebrates: such as ants, beetles and other animal material; (3) Feathers; and (4) Hair and other non-identifiable material.

### Stable isotope analysis

Isotopic signatures of δ^15^N and δ^13^C were measured from rodents’ hair and from tissues of their potential food sources. Hair was collected from the dorsum of 40 black rats, 4 Norwegian rats and 16 mice captured at the study sites. This tissue allows inferences about the diet of an organism on a temporal scale of weeks (~40 days in *R*. *norvegicus*) [30], because it is metabolically inert and therefore reflects the diet of individuals during a limited period of tissue growth [31].

Plant and terrestrial invertebrate samples were collected in the proximity of the sampling stations located in El Junco. The leaves of different plant species (three individuals per species) were collected in paper bags and conserved by refrigeration for up to 12 hours until being processed in the laboratory. Diverse species of terrestrial invertebrates (five individuals per species) such as arthropods and mollusks were collected in 1 x 1 m quadrants placed in various sections of the transect where the traps where located, mainly where the highest rodent capture was recorded. The collected organisms were stored in 80% alcohol in plastic containers. Petrel feathers (contour feathers) were collected from 16 nests inspected in the four study colonies and stored in paper bags.

Fur and feather samples were cleaned of surface contaminants using a chloroform/methanol solution (1:1) to extract lipids. This process was applied because these samples contain appreciable quantities of ^13^C-depleted lipids [31]. The samples were air-dried and then cut into very small pieces [32]. Next, they were ground to a fine powder, and then were sealed (∼1 mg) in 8 x 5 mm tin capsules. Leaves and complete invertebrate specimens were dried at a temperature of 60°C for 24 hours [33], and then were pulverized. Later, the samples (1 mg for animal tissues and 5mg for plant tissues) were sealed in 8 x 5 mm tin capsules for isotopic analysis.

Isotope ratios were measured under continuous flow in a mass spectrometer (20–20 PDZ Europe, Sercon Ltd., Cheshire, UK) at the Stable Isotope Facility, University of California at Davis. The results are presented in parts per thousand (‰) using the following equation: δ^15^N or δ^13^C = ((R _sample_ / R _standard_) -1) * 1000‰, where, R _sample_ and R _standard_ are the values of ^15^N/ ^14^N or ^13^C/ ^12^C in the sample and standard, respectively. The standards were atmospheric N_2_ for nitrogen and Pee Dee Belemnite (PDB) for carbon. The results were calibrated with international standards (ammonium sulfate for δ^15^N and sucrose for δ^13^C), which generated a standard deviation between the isotopic measurement trials of < 0.3‰ for δ^15^N and < 0.2‰ for δ^13^C.

To construct the trophic structure of *R*. *rattus* from El Junco, average isotopic values estimated for plant and invertebrate species collected in the field and identified as prey from the rat’s stomach contents analysis were used. Because the factors of isotopic discrimination depend on multiple sources of variation such as taxon, environment or tissue, discrimination factors (Δ^13^C and Δ^15^N) for the rat hair were calculated from specific regression equations between discrimination factors of the black rat’s hair and corresponding isotopic proportions of its prey, as proposed by [30] and applied by [34]: Δ^15^N_rat’s hair-diet_ = 0.05 * (δ^15^N_prey_)^2–^0.94 * δ^15^N_prey_ + 3.18; Δ^13^C_rat’s hair-diet_ = -0.66 * δ^13^C_prey_—14.41.

### Data analysis

Population abundance of rodent species per study site was estimated by the capture success, which is defined as the number of captured individuals per unit of time and effort, expressed as a percentage.

The frequency of occurrence (number of stomachs that contained each item expressed in percentage) was calculated from the stomach content analysis of *R*. *rattus* and *R*. *norvegicus*, and the relative abundance of each type of food was calculated for each individual. This analysis was performed using a 5 x 5 mm grid under the Petri dish that contained the remnants of food from each stomach, and the number of squares containing each type of food was quantified and divided by the total number of squares in the grid that contained food. The relative abundance value for each food was expressed as a percentage.

After prior testing for the normality and homoscedasticity of the data, the Kruskal-Wallis test was used to identify differences in relative abundance of prey consumed by *R*. *rattus* in El Junco, San Joaquín and La Comuna. Isotopic differences in hair between rodent populations from one site and among sites were evaluated using the Kruskal-Wallis test followed by a multiple comparison of mean ranks for all the groups. The analyses were conducted using Statistica version 8.0 (StatSoft. Inc., Tulsa, OK, U.S.A). Statistical significance was assumed at a value of *P* <0.05.

## Results

### Population abundance

The black rat (n = 43) and house mouse (n = 17) were collected at all four sites, whereas the Norwegian rat (n = 4) was only captured at La Comuna. The rats were dominant in captures (average capture percent (ACP) = 73.6%) compared to mice (ACP = 26.4%), which had a capture rate between 25% and 33% compared with the 69% to 77% recorded for rats.

Three specimens of *R*. *rattus* collected corresponded to the subspecies *frugivorous* and were captured in El Junco (two individuals) and La Comuna (one individual), and the remaining individuals corresponded to the subspecies *R*. *r*. *rattus*. The identification was made on the basis of morphological characteristics of the subspecies. The hair color on the dorsum of *R*. *r*. *rattus* is black or dark gray and of a lighter shade on the abdomen; by contrast, *R*. *r*. *frugivorous* has a lighter and smaller body build than the other subspecies, and its hair color is reddish-brown on the dorsum and creamy white on the abdomen and throat [35].

The trapping effort for each site differed due to the number of active traps per night and the number of sampling periods. In the colonies of La Comuna, El Junco and San Joaquín, three capture nights were conducted, and the calculated effort was 88, 87 and 89 active traps, respectively for rats; and 56, 55 and 56 active traps, respectively for mice. In El Plátano, two capture nights were conducted, and the calculated effort was 37 and 11 active traps for rats and mice respectively. The abundance of each species per site estimated by the capture success is shown in Table 1.

**Table 1:** Number of rodents captured in four nesting colonies of Galapagos petrel on San Cristóbal Island during sampling in June 2013.

Species	Colonies				
	El Plátano	El Junco	San Joaquín	La Comuna	Total by species
*R*. *rattus*	3 (8.1%)	10 (12.6%)	11 (12.4%)	19 (22.7%)	43
*R*. *norvegicus*	0	0	0	4 (4.5%)	4
*M*. *musculus*	1 (9.1%)	5 (9.1%)	4 (7.1%)	7 (12.7%)	17
**Total by colony**	4	15	15	30	64

The capture success (%) per species and site is shown in parenthesis.

### Stomach contents

In total, 47 individuals of the genus *Rattus* were dissected. An empty stomach and seven containing only nematodes were discarded. Nematodes are parasites of arthropods or other terrestrial invertebrates that entered the rodents by consuming these hosts and therefore do not form part of their diet.

From the 39 analyzed stomachs (*R*. *rattus*: n = 37; *R*. *norvegicus*: n = 2), two main types of food were identified: Plants, from which seven species were identified, and invertebrates, including five distinct groups of species. Hair and non-identified material (highly digested), found in 89% of the stomachs, formed a third category. The intake of hair is due to frequent grooming by the rats and may average 5% of the volume of stomach contents [16]. A non-identified nematode species was present in 72.5% of all stomachs. No feathers of petrels or any other bird were found in stomachs.

**Fig 2:**
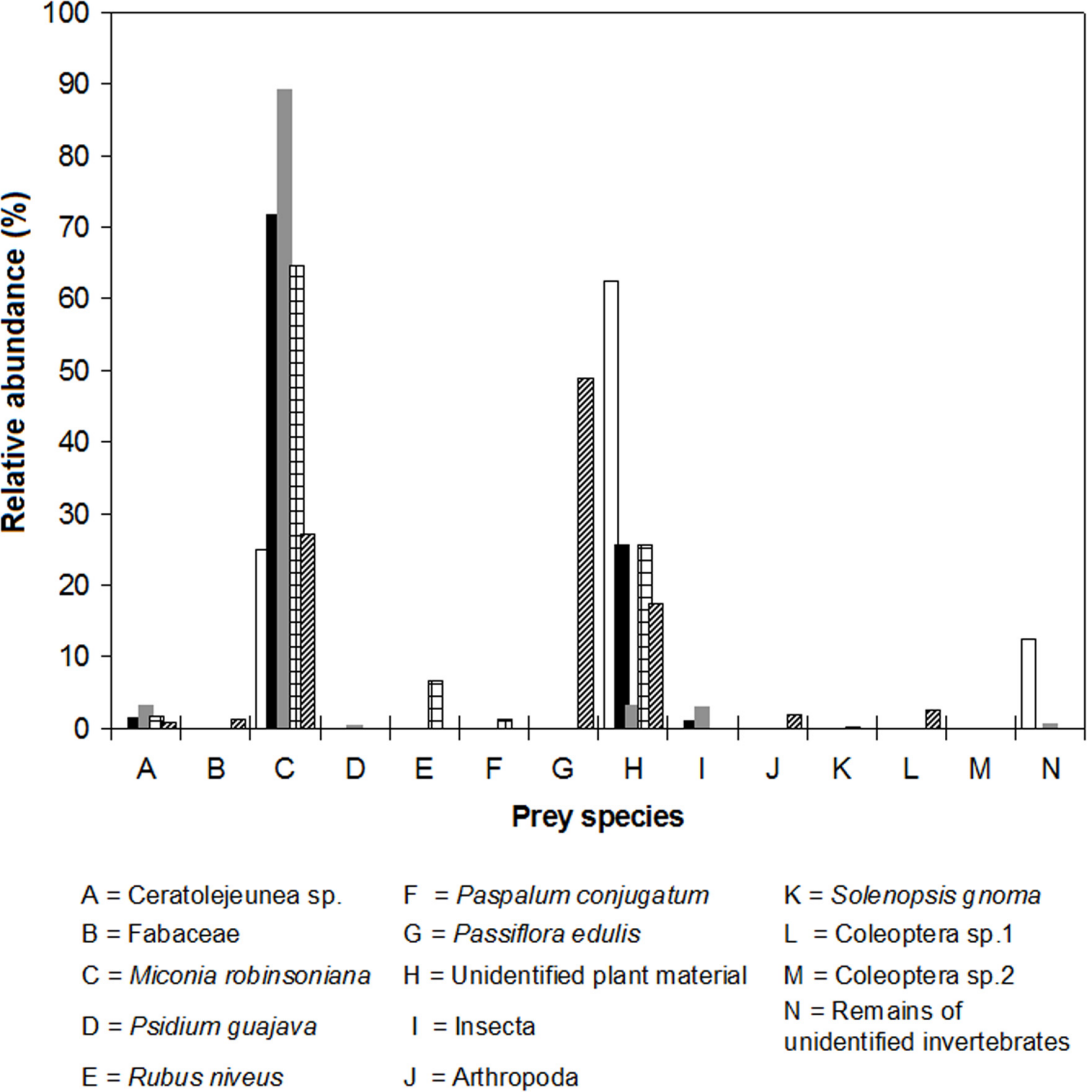
Relative abundance (%) of prey species and other food items found in stomachs of black rats collected during June 2013 in four nesting colonies of Galapagos petrel on San Cristóbal Island: El Plátano (white bars), El Junco (black bars), San Joaquín (gray bars), La Comuna (plaid bars). It is also shown the relative abundance of preys found in stomachs of Norwegian rats (striped bars) collected in La Comuna.

In general, the diet of *R*. *rattus* was composed primarily of plants (relative abundance (RA) = 98.20%), and plant material was present in all the stomachs. Animal material represented 1.80% of RA and was found in 81% of the analyzed stomachs. The endemic shrub *Miconia robinsoniana* was the most frequent (95%) and abundant food (RA = 72.10%) in the stomachs of the four sites (Fig 2, Table 2). No significant differences in relative abundance of consumed prey by *R*. *rattus* were found in El Junco, San Joaquín and La Comuna (Kruskal-Wallis: H_(2,42)_ = 0.75, *P* = 0.688). Data from the one rat caught at El Plátano were not included.

**Table 2:** Percentage of frequency of occurrence (FO) and relative abundance (RA) of prey species and other food items found in the stomachs of *R*. *rattus* and *R*. *norvegicus* (last column) collected in nesting colonies of Galapagos petrel on San Cristóbal Island during June 2013.

Division-Class/Family	Prey species	El Plátano		El Junco		San Joaquín		La Comuna		Total—*R*. *rattus*		*R*. *norvegicus*	
		(n = 1)		(n = 10)		(n = 10)		(n = 16)		(n = 37)		(n = 2)	
		FO	RA	FO	AR	FO	RA	FO	RA	FO	RA	FO	RA
	**Vegetal material**		**87.50**		**98.90**		**96.10**		**99.70**		**98.20**		**95.50**
Briophyta (Hepaticopsida)	*Ceratolejeunea sp*.◊	0	0.00	20	1.50	40	3.15	25	1.69	27	1.99	50	0.71
Magnoliophyta (Magnoliopsida)	Fabaceae	0	0.00	0	0.00	10	0.08	0	0.00	3	0.02	50	1.34
Magnoliophyta (Melastomataceae)	Miconia robinsoniana*	100	25.00	90	71.78	100	89.21	94	64.56	95	72.10	100	27.21
Magnoliophyta (Myrtacea)	*Psidium guajava* ◊	0	0.00	0	0.00	20	0.43	0	0.00	5	0.12	0	0.00
Magnoliophyta (Rosaceae)	*Rubus niveus †*	0	0.00	0	0.00	0	0.00	50	6.62	22	2.86	0	0.00
Magnoliophyta (Poaceae)	*Paspalum conjugatum †*	0	0.00	0	0.00	0	0.00	6	1.25	3	0.54	0	0.00
Magnoliophyta (Passifloraceae)	*Passiflora edulis* ◊	0	0.00	0	0.00	0	0.00	0	0.00	0	0.00	50	48.81
	Unidentified plant material	100	62.50	90	25.64	60	3.25	69	25.63	73	20.58	50	17.38
	**Animal material**		**12.50**		**1.10**		**3.90**		**0.30**		**1.80**		**4.50**
Arthropoda (Insecta)	Insecta	0	0.00	20	1.04	30	3.10	0	0.00	14	1.12	0	0.00
Arthropoda	Arthropoda	0	0.00	0	0.00	10	0.08	0	0.00	3	0.02	50	1.87
Arthropoda (Formicidae)	Solenopsis gnoma*	0	0.00	0	0.00	0	0.00	13	0.25	5	0.11	0	0.00
Arthropoda (Coleoptera)	Coleoptera sp.1	0	0.00	0	0.00	0	0.00	0	0.00	0	0.00	50	2.67
Arthropoda (Coleoptera)	Coleoptera sp.2	0	0.00	10	0.04	0	0.00	0	0.00	3	0.01	0	0.00
	Remains of unidentified Invertebrates	100	12.50	0	0.00	10	0.70	0	0.00	5	0.53	0	0.00

For *R*. *rattus*, the percentage values per site and total are shown. The sample size in each case is shown in parentheses. *R*. *norvegicus* was only captured in La Comuna.

*Endemic species in the Galapagos Islands.

◊ Native species in the Galapagos Islands.

† Introduced species in the Galapagos Islands.

### Stable isotopes

The δ^13^C and δ^15^N signatures from the hair of 60 rodents (*M*. *musculus*: n = 16, *R*. *rattus*: n = 40, *R*. *norvegicus*: n = 4) were analyzed. No significant difference was found in δ^13^C among species (Kruskal-Wallis: H_(2,60)_ = 3.14, *P* = 0.208). The δ^15^N values were significantly different (Kruskal-Wallis: H_(2,60)_ = 7.56, *P* = 0.023) between *R*. *rattus* and *M*. *musculus* (Multiple Comparisons of mean ranks: *P* = 0.036).

**Fig 3:**
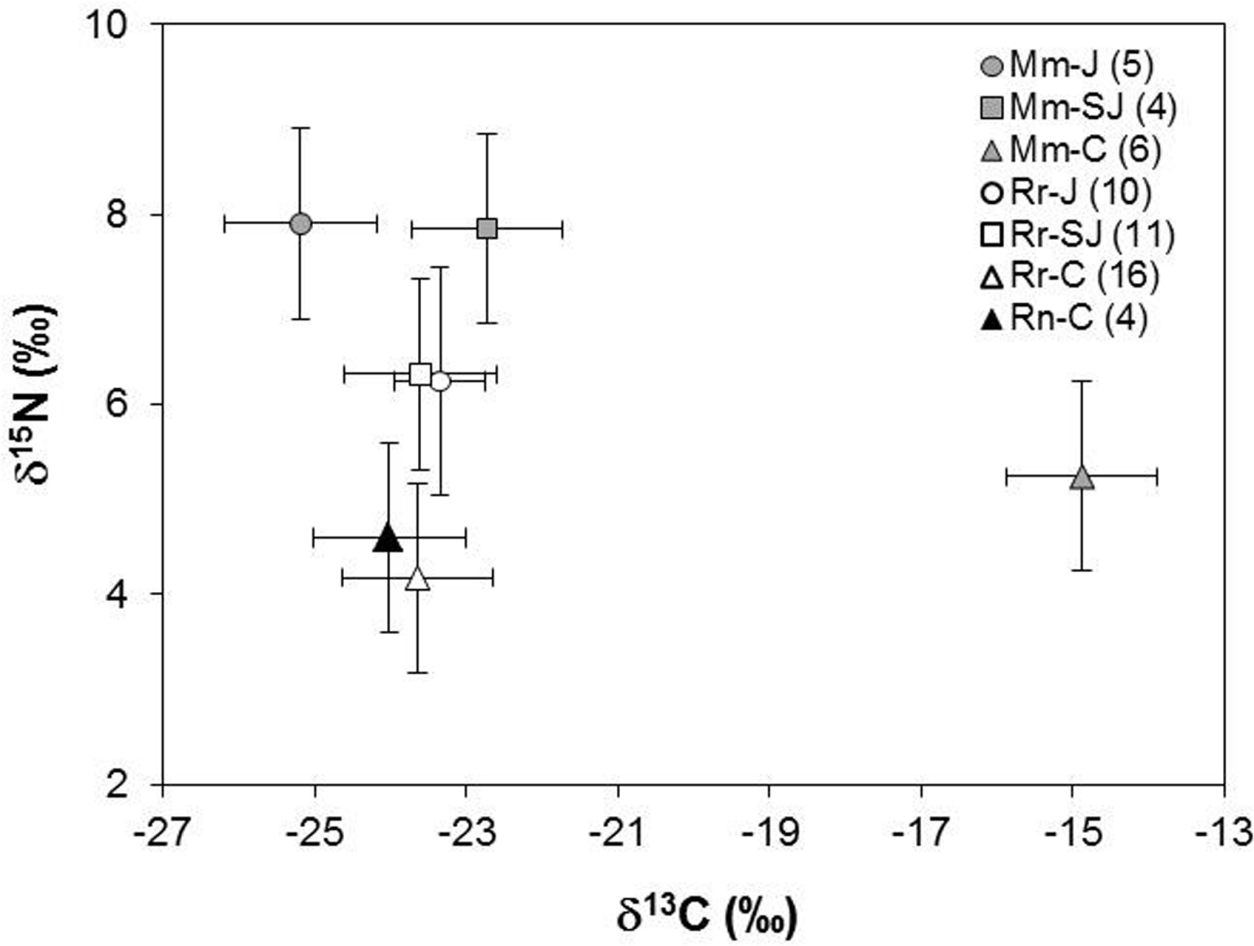
Means (± SD in ‰) δ13C and δ15N values in fur of M. musculus (gray simbols), R. rattus (White symbols) and R. norvegicus (black symbol) collected during June 2013 in three breeding colonies of Galapagos petrel on San Cristóbal Island: El Junco (J) (circles), San Joaquín (SJ) (squares) and La Comuna (C) (triangles). The sample size for each species per collection site is shown in the parentheses besides the code of the species and the initial of the study site. Mm = *M*. *musculus*; Rr = *R*. *rattus*; Rn = *R*. *norvegicus*.

At intraspecific level (Fig 3), *M*. *musculus* showed significant differences in δ^13^C among sites (Kruskal-Wallis: H_(2,15)_ = 10.08, *P* = 0.006). The multiple comparisons of mean ranks determined that La Comuna was statistically different of El Junco (*P* = 0.006). For δ^15^N, no significant differences were found among the compared populations (Kruskal-Wallis: H_(2,15)_ = 5.06, *P* = 0.079). By contrast, for *R*. *rattus*, the δ^13^C values were not significantly different among sites (Kruskal-Wallis: H_(2,37)_ = 2.56, *P* = 0.277), but for δ^15^N, significant differences (Kruskal-Wallis: H_(2,37)_ = 2.56, *P* = 0.277) were found between La Comuna and El Junco (Multiple Comparisons of mean ranks: *P* = 0.002) and between La Comuna and San Joaquín (Multiple Comparisons of mean ranks: *P* = 0.001). El Plátano was out of this analysis because this site has not an adequate sample size.

**Fig 4:**
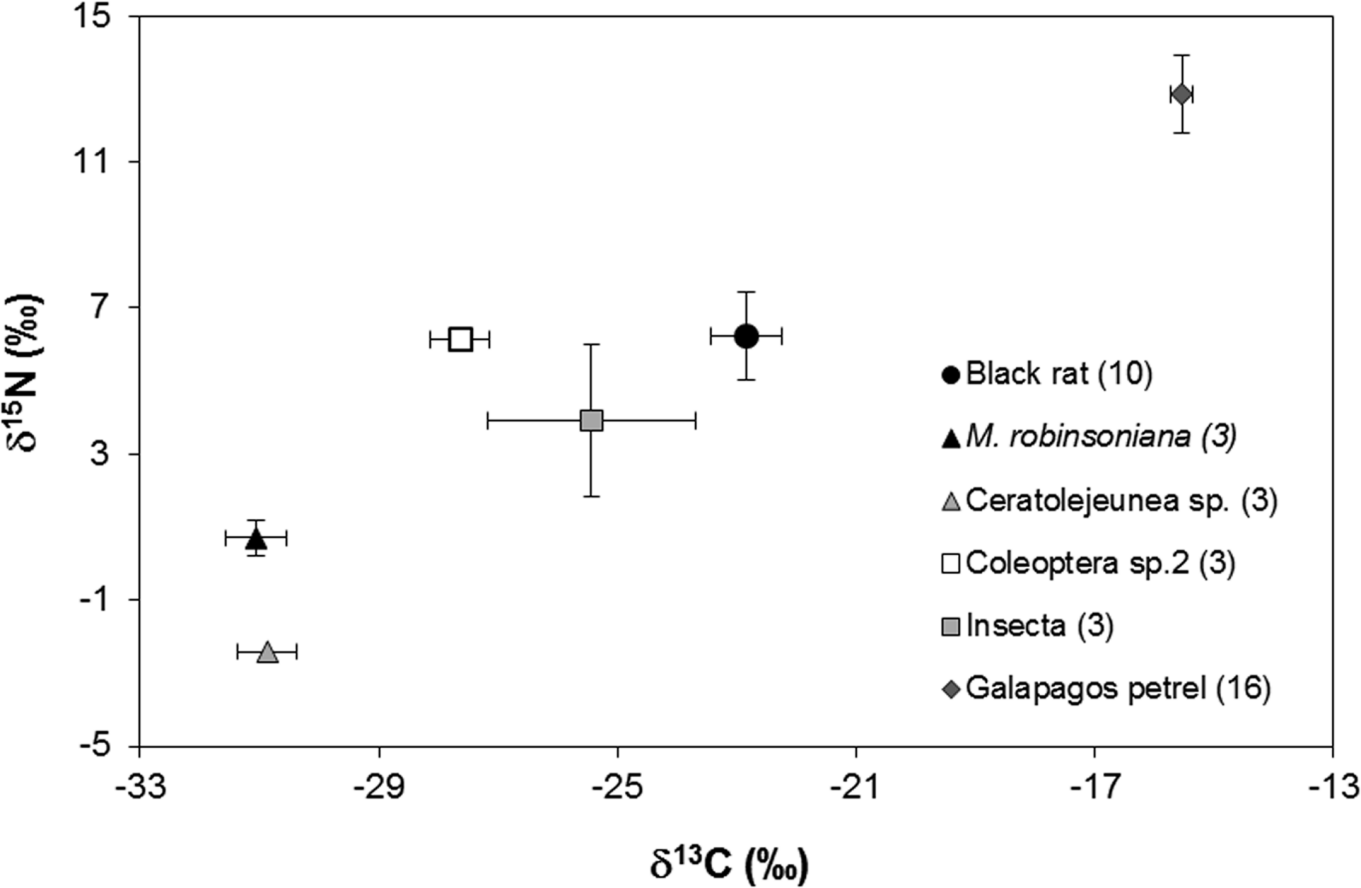
Means (± SD in ‰) δ^13^C and δ^15^N values in fur of black rats and their prey identified by the stomach contents analysis. The samples were collected in El Junco during June 2013. The sample size for each species is shown in parentheses.

The trophic structure of *R*. *rattus* from El Junco is provided in Fig 4. The isotopic discrimination factor (Δ^13^C and Δ^15^N) calculated between *R*. *rattus* (Rr) and their prey was as follows: Rr-*Ceratolejeunea sp*.: Δ^13^C = 5.96‰, Δ^15^N = 5.75‰; Rr-*M*. *robinsoniana*: Δ^13^C = 6.09‰, Δ^15^N = 2.54‰; Rr-Coleoptera sp. 2: Δ^13^C = 3.83‰, Δ^15^N = -0.71‰; Rr-Insecta: Δ^13^C = 2.24‰, Δ^15^N = -0.08‰. Although no feather remains were found in the rat’s stomachs, the isotopic signatures of petrel feathers and black rat hair were compared. A difference of more than 7‰ in δ^15^N and 6‰ in δ^13^C was found, which indicates that the rodents did not consume petrel during the approximately 40 days prior to their capture.

## Discussion

The presence of black rat and house mouse has been recorded in petrel nesting colonies on several islands [8,27]. Nonetheless, this study is the first record of the Norwegian rat and the subspecies of black rat *R*. *r*. *frugivorous* at these sites on San Cristóbal Island.

The population of black rats was dominant in abundance compared to the house mouse in the four sites monitored. However, the abundance estimated for the two sympatric populations may be underestimated. Although we used a lower trapping effort for the house mouse, it has been shown that the number of captures of house mice increases once the catch rate of black rat substantially decreases [12]. In our case, the trapping events were simultaneous for both species by study site, and thus, the activity and numbers of *M*. *musculus* may be suppressed by *R*. *rattus*.

The black rats eat a broad range of plant and animal food types; nevertheless, they are highly selective feeders [3]. The combination of two or three food types is selected by rats in their diet, with one of them being more frequent and abundant [13]. Plant material often comprises 75–80% of the diet of black rats in the Pacific islands [26], and fruits and seeds are the most common plant items in their diet [5]. Arthropods, particularly insects, are an important dietary component of most black rats, but they typically comprise a smaller component of the *R*. *rattus* diet relative to vegetative material [16].

The diet of black rats was 98% plant material, the seeds of *Miconia robinsoniana* being dominant, and only 2% arthropods. These results are consistent with those found by [3] in the montane shrub ecosystem in Santa Cruz Island, Galapagos; where almost all rats ate Miconia fruit, Blechnum sp. rhizomes and arthropods.

Black rats’ diets differ greatly among habitats and between seasons within a habitat [3]. The sites of our study correspond to the Miconia zone (400–700 m asl). Although the food items identified in the black rats’ stomachs and their relative abundance were similar among sites, a broader diet in La Comuna was found, with the dominance of Miconia seeds persisting but at a lesser percentage (65%) compared to El Junco (72%) and San Joaquín (89%). This variability may be related to the lower density of Miconia shrubs in La Comuna compared to the other sites, the latter two being closer to each other (about 3683 m apart), with approximately equivalent elevation (El Junco: 620 m, San Joaquín: 637 m) and with similar environmental characteristics.

The consumption of Miconia fruits by black rats is subject to its abundance in the habitat, which changes between seasons [3]. Miconia commonly flowers from February to March, producing small berries containing hundreds of tiny seeds (1 mm in size). The dispersion of various native and non-native species seeds by black rats has been demonstrated in several islands [25,36,37,38]. The majority of the seeds that are dispersed by black rats are small (<1.5–2.2 mm) and survive ingestion and gut passage [36,37].

According to [3] and [17], *R*. *rattus* tend to be seed predators rather than dispersers in the Galapagos Islands, because the seeds ingested were destroyed and unable to germinate. Conversely, in our study, almost all the Miconia seeds found in the stomachs were intact, showing that black rats might play an important role as seed dispersers in the archipelago.

The Norwegian rats were suggested to be significant seed dispersers in the Galapagos Islands, based on germination studies of recovered seeds from their scats [17]. In our study, Miconia seeds were the second most common food item found in the Norwegian rats’ stomachs. Although all the seeds were intact, this result was based on only two individuals analyzed, and further research is required to strengthen this hypothesis. Additionally, our results are the first record of the *R*. *norvegicus*’ diet in the Galapagos Islands in the wild.

Bird eggs and feathers in black rats’ stomachs are evidence that either juvenile or adult birds have been consumed directly or scavenged [3,39,40]. Nonetheless, the consumption of birds by rats seems to only supplement their diet [28,32], with some exceptions during the seabird nesting season [24].

On San Cristóbal Island, the petrel population has a prolonged reproductive period covering 10 months. Laying dates occurred mostly from May to October, with a peak during August, although eggs may be occasionally laid between November and March [27]. Trapping was conducted during June when Miconia fruits were abundant and available to rats. The 26% of nests monitored contained chicks, so it was expected that predation on petrel might not be significant.

None of the evidence from stomachs in this study showed that rats ate birds. Nor were petrel carcasses observed near their nesting burrows. In 2011, nine predated petrel chicks were found in the study colonies by the Galapagos National Park (GNP) rangers during the regularly programmed monitoring of petrel nesting zones, but in 2012 and 2013, no predation was reported (GNP, pers. comm.). This suggests that rats might not consume petrel as a complementary prey even when their preferred food is not so available in their habitat. Nevertheless, a further long-term study is necessary to test this hypothesis. Therefore, the consumption of petrel by rats cannot be completely discard.

The isotopic values from fur of rodents were consistent with the stomach contents analysis results, showing that the rodents’ diet is based on plants and arthropods. The consumption of petrel chicks during 40 days prior to rodents’ capture was not evidenced by the stable isotope analysis (SIA) on feathers; however, the SIA on egg shells might clarify if the predation impact of rats on petrels is rather focused on their eggs. Anyway, these results should be interpreted with caution because they reflect the diet of rats during a brief period of the year,

The δ^13^C and δ^15^N signatures between rodents’ species reflect, in general, that *R*. *rattus* feeds in a lower trophic position than *M*. *musculus*. This is consistent with the analyses of stomach contents of black rats, in which plant matter was the principal component of their diet, represented to a great extent by seeds and fleshy fruits. On the other hand, diet studies in Hawaii and Southern Ocean islands demonstrate that the house mouse, although it consumes plants, is more carnivorous than the black rat. Fruits form only 10–11% of the mouse’s diet [14,16], and arthropods account for an average of 57% of their stomach contents [15].

In the comparison of the isotopic signals from the three coexisting rodents in La Comuna, the house mouse showed higher isotopic values than rats. Norwegian rat also showed average δ^13^C and δ^15^N values slightly lower and higher, respectively, than black rat. This reflects the differential use of diet resources by rodents’ species, due to the unequal consumption of prey species that occupy different trophic levels [16].

This was evidenced in the stomach contents analysis which showed similarities in the diet of black rats and Norwegian rats. Certain food items were common in their diets but consumed in different percentages, whereas, other food items were particular to each one of rats’ species. Nonetheless, further studies are recommended to determine the possible variability in the rats’ diets over time, including its relationship to their reproduction cycle and the variability in the abundance of their prey at these sites.

At intraspecific level, the *M*. *musculus* populations of El Junco and San Joaquín had δ^13^C and δ^15^N signatures (although not significant for δ^15^N) that were different from those at La Comuna. In *R*. *rattus*, such differences were only evident in δ^15^N among the populations of El Junco and San Joaquín compared to La Comuna.

Elevational gradients in plant δ^13^C values have been demonstrated by several authors and are thought to be associated with plant physiological adaptations to changes in growing conditions and *p*CO_2_ with elevation [41]. A slight enrichment in plant ^13^C content with elevation by a few per mil over 1000 or more meters has been demonstrated [41,42]. There is limited evidence that soil δ^15^N values may become more negative with elevation at a given location [43], and that these patterns may also be reflected in local food webs [44].

The sites of our study are at different elevations but in the same vegetation region (between 400 and 700 m). Therefore, it is unlikely that elevational gradients affect the isotopic values of the food webs. Instead, δ^13^C differences among house mouse populations likely involve unequal consumption of C3 and C4 plants [45]. This may even explain the higher δ^13^C values of *M*. *musculus* in La Comuna compared to the other populations of rats and house mouse. In La Comuna, the majority of C4 plants are grasses such as *Paspalum conjugatum*, and some of the mice captured were near these grasses. The δ^13^C signals of mice in La Comuna (-14.4 ‰) were approximately 10 ‰ higher than those of mice from El Junco (-24.7 ‰) and San Joaquín (-22.2 ‰). Although we cannot account for this difference with dietary information from house mouse stomach contents, a previous study suggests that this species consumes C4 plants in insular habitats [16].

Differences in δ^15^N values among black rat populations likely reflect a broader diet in La Comuna compared to El Junco and San Joaquín. This is based on the stomach contents, which showed that black rats in La Comuna ate Miconia as the main prey and complemented their diet with three other items (*Rubus niveus*, *P*. *conjugatum* and *S*. *gnoma*). This foraging behavior appears to be related to the reduced density of Miconia shrubs at La Comuna and the ability of black rats to adapt their diet to the resources available in their habitat.

The results of this study contribute not only to the understanding of the trophic ecology of black rats in an insular environment but also to their relationship with the two other introduced rodents in the Galapagos Islands, as well as the possible impacts on particular flora and fauna species of the archipelago. These findings may be useful when establishing future management priorities for the introduced and endemic species of the archipelago.
